# The advantages of microfluidics to study actin biochemistry and biomechanics

**DOI:** 10.1007/s10974-019-09564-4

**Published:** 2019-11-20

**Authors:** Hugo Wioland, Emiko Suzuki, Luyan Cao, Guillaume Romet-Lemonne, Antoine Jegou

**Affiliations:** Institut Jacques Monod, CNRS, Université de Paris, 75013 Paris, France

**Keywords:** Actin, Microfluidics, Microscopy, Cofilin, Formin

## Abstract

The regulated assembly of actin filaments is essential in nearly all cell types. Studying actin assembly dynamics can pose many technical challenges. A number of these challenges can be overcome by using microfluidics to observe and manipulate single actin filaments under an optical microscope. In particular, microfluidics can be tremendously useful for applying different mechanical stresses to actin filaments and determining how the physical context of the filaments affects their regulation by biochemical factors. In this review, we summarize the main features of microfluidics for the study of actin assembly dynamics, and we highlight some recent developments that have emerged from the combination of microfluidics and other techniques. We use two case studies to illustrate our points: the rapid assembly of actin filaments by formins and the disassembly of filaments by actin depolymerizing factor (ADF)/cofilin. Both of these protein families play important roles in cells. They regulate actin assembly through complex molecular mechanisms that are sensitive to the filaments’ mechanical context, with multiple activities that need to be quantified separately. Microfluidics-based experiments have been extremely useful for gaining insight into the regulatory actions of these two protein families.

## Introduction: some challenges of studying actin assembly dynamics

The various actin filament networks in cells are responsible for a number of important processes. Their diverse architectures and turnover rates are tightly regulated in order to generate the proper filament organization at the right time and place (Campellone and Welch 2010; Blanchoin et al. 2014; Skau and Waterman 2015; Plastino and Blanchoin 2019). This is orchestrated by a large array of actin binding proteins (ABP), which are able to perform various actions on actin monomers and filaments (Pollard 2016). They may, for example, assist or prevent the nucleation of new filaments, regulate their elongation from either end, crosslink filaments together, stabilize or sever filaments, and regulate their disassembly from either end. When a new ABP is discovered, the first task is thus to determine what it actually does to actin, i.e. to identify biochemical reactions and to quantify their rates. This task is complicated by the possibility for a protein to combine different actions, which may be difficult to disentangle.

In the cell context, ABPs do not act independently. Another challenge is thus to determine how different ABPs modify each other’s actions, i.e. how they compete or synergize. In addition to this, other factors modulate ABPs’ interactions with actin, such as the filament’s nucleotide state, its post-translational modifications, and even its mechanical environment (Jégou and Romet-Lemonne 2016; Harris et al. 2018). Experiments thus need to be quantitative and to be repeated under different biochemical and mechanical conditions.

Addressing these challenges requires the ability to monitor individual events, on individual filaments. This requirement raises challenges of its own. The first is that one does not learn much by observing an individual event a few times. Large amounts of data have to be collected in order to reliably determine reaction rates. The second is that observing individual filaments live mostly relies on fluorescence microscopy techniques and requires binding filaments to surfaces or constraining their movements to allow their observation over time. These technical strategies (labeling, binding…), like any other, can alter the parameters one is trying to measure. Potential artefacts need to be tracked down, systematically and carefully.

Microfluidics has emerged as a versatile technique for a number of experimental situations, in different fields of research. Over the past 10 years, it has been applied to the manipulation and observation of individual actin filaments, in order to study the regulation of their assembly dynamics.

In the following sections, we will present this method (“Simple microfluidics for the study of individual actin filaments” Section) and its main assets (“Key advantages for the study of actin assembly dynamics” Section), which help to address most of the challenges we have listed above. We will then go over concrete examples with the study of formins (“Using microfluidics to study rapid filament elongation by formins” Section) and ADF/cofilin (“Using microfluidics to study the disassembly of actin filaments by ADF/cofilin” Section), for which microfluidics has been extremely useful.

## Observing and manipulating actin filaments with the help of microfluidics

### Simple microfluidics for the study of individual actin filaments

“Microfluidics” generally refers to a technique where fluids are flowed through micrometer-size channels. Studying the properties of flowing fluids at such small scales is a very active research field of its own (Squires and Quake 2005). Tremendous progress of this field over the past decades has led to a broad range of applications. In the life sciences, in particular, microfluidics is now commonly used in a number of experiments (Sonnen and Merten 2019), including the creation of concentration gradients in which cells migrate (Toh et al. 2014), the application of mechanical stresses to living cells (Kurth et al. 2012), and the generation of droplet-size reactors in which to encapsulate reagents (Seemann et al. 2012). Beyond basic research, microfluidics is also a powerful tool for DNA manipulation (Wu et al. 2014) and drug screening (Wu et al. 2010).

In this review, we focus on the benefits of microfluidics for the study of individual actin filaments. The general purpose of the technique is to ease the manipulation and the imaging of these filaments by light microscopy, in order to gain insight into various processes, both biochemical and mechanical, which control actin assembly dynamics. Compared to other applications of microfluidics, such as the examples cited in the previous paragraph, this is a fairly simple and affordable experimental configuration, readily accessible to non-specialists. Recent developments in commercially-available microfluidics devices also make it easy to implement this technique in virtually any lab.

**Fig. 1:**
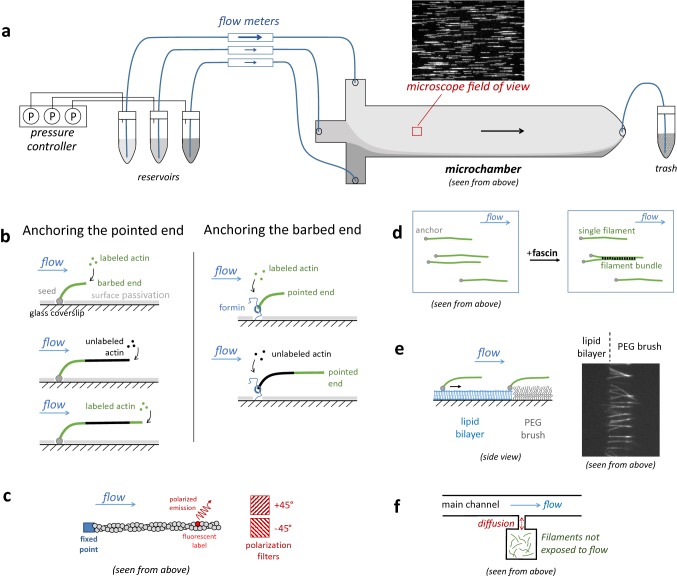
Microfluidics setup for the study of individual actin filaments. **a** Sketch of the setup, with a microscope image (epifluorescence, image width 83 µm) of a typical field of view, showing filaments anchored by their pointed end, on the left hand side. The pressures in the three solution reservoirs are controlled in order to modify the incoming flow rates. Typical dimensions of the main channel, in the microchamber: 800 µm wide, 1 cm long, 40 µm high. The chamber is sketched seen from above. **b** Sketches (side view) illustrating different anchoring strategies. In each configuration, a significant tension can be applied to the filament and its anchoring point. Left: filaments are anchored by their pointed ends (spectrin-actin seed adsorbed to the surface). Right: filaments grow from their anchored barbed ends (formin bound to the surface). Alternatively, stabilized barbed ends can be anchored, using biotinylated gelsolin for example. In each configuration, alternating the incoming solutions allows one to generate unlabeled actin segments. **c** Thanks to the polarization of the light emitted by the fluorophore bound to an actin subunit, one can monitor the orientation of that subunit around the filament axis. **d** Neighboring filaments can form bundles when exposed to bundling proteins such as fascin. **e** Filaments anchored to a lipid bilayer are dragged to the edge of the bilayer by the flow. Left: sketch, from a side view. Right: epifluorescence microscopy image showing filaments gathered at the edge of a lipid bilayer and bundled by fascin. Filaments are approximately 10 µm long

Our basic, single actin filament experimental configuration using microfluidics exploits the fact that actin filaments are semi-flexible and align with the flow when they are anchored by one end (Jégou et al. 2011b). This can be achieved by growing filaments from surface-anchored seeds (i.e. short, stabilized filaments) or by attaching them to an anchored end-binding protein, while the surface is otherwise passivated. When filaments are long enough (typically, a few micrometers or more) and the flow velocity high enough (typically, 10 µm/s or more, close to the surface) the filaments are maintained within a few hundred nanometers above the surface and align with the flow (Fig. 1). This is directly inspired by work on DNA using microfluidics, with the notable difference that DNA is a far more flexible filament and thus spontaneously coils up in the absence of flow and is uncoiled by the flowing solution (Brewer and Bianco 2008). Microtubules, which are stiffer than actin filaments, can also be studied in a similar fashion using microfluidics (Schaedel et al. 2015; Duellberg et al. 2016).

Note that another use of microfluidics, where non-anchored single filaments are tracked as they flow down the channel, also offers interesting possibilities, for example as a means to impose mechanical stress on the filaments (Köster et al. 2005; Liu et al. 2018). They will not be discussed here, and hereafter, we focus on situations where filaments are anchored to the bottom surface of the flow chamber.

One of the great benefits of microfluidics is the possibility to rapidly change the solution to which the anchored filaments are exposed, by using chambers with multiple entries. (Figure 1a, b). In the main channel, the different incoming solutions flow side by side, with neat boundaries and virtually no mixing (laminar flows). Today’s pressure control devices can rapidly change the pressures applied in the solution reservoirs, thereby modulating the incoming flow rates and thus moving the boundaries between the flowing solutions inside the main channel. This allows the exchange of the solution to which the anchored filaments are exposed (in the microscope field of view) in less than one second.

The flowing solution exerts a viscous drag force on the anchored filaments. As a result, filaments align with the flow, as already mentioned, and a tensile stress is applied to the filaments. This tension is not uniform, but gradually increases from the free end where it is negligible, to the anchoring point where it is maximal. This force can be calibrated based on the drag coefficient of actin filaments, the flow rate profile, and the filament contour height as it fluctuates above the coverslip surface (Jégou et al. 2013; Wioland et al. 2019a). By controlling the flow rate, the tension applied to the filaments and their anchoring point can be modulated, up to a few tens of picoNewtons. Note that it is possible to apply negligible tension to filaments (less than 0.1 pN) with very low flow rates which are nonetheless enough to align the filaments for proper imaging. It is thus possible to use microfluidics to study actin dynamics and benefit from all its assets (see “Filaments appear straight and parallel to the direction of the flow, easing analysis” Section) without applying significant forces to the filaments.

Technically, the basic experimental configuration we have described here is not very demanding. It has some specific equipment requirements related to the use of microfluidics: pressure controllers and flow meters (commercially available) and the microchamber itself, which can be made in the lab (usually using polydimethylsiloxane and a mold with the desired channel pattern, which can itself be made in the lab or purchased from a specialized company) or bought ready-made. The chamber height is typically a few tens of micrometers. The other technical requirements are very similar to those of classical single-filament observations that do not employ microfluidics. In particular, glass surfaces must be properly cleaned, passivated and functionalized.

Other experimental configurations can be achieved, as simple variations of the basic setup we have described. For example, while the flow lines are practically parallel to the direction of the main channel downstream of the entry channels’ junction, this is not the case in the vicinity of the junction itself. This region can be exploited in order to modulate the direction in which the filaments align. Also, rather than anchoring filaments by one point only, one can purposely anchor filaments at multiple sites (Wioland et al. 2019a). In addition, the setup can become more sophisticated as microfluidics can be combined with other techniques (see “Microfluidics can be combined with other techniques” Section for more examples).

### Key advantages for the study of actin assembly dynamics

In this section, we highlight what we believe to be the main assets of microfluidics for the study of individual actin filaments. In the next sections, we give examples of how these advantages concretely play out, by going through recent results obtained thanks to microfluidics on two key protein families: formins, which can rapidly generate long actin filaments (“Using microfluidics to study rapid filament elongation by formins” Section), and proteins of the ADF/cofilin family, which are the central players in actin filament disassembly (“Using microfluidics to study the disassembly of actin filaments by ADF/cofilin” Section).

#### Filaments are anchored by a single point, limiting the risk of artefacts

Single filament studies require that filaments be maintained close to the surface in order to image them, in particular with total internal reflection microscopy (TIRFM). This is classically achieved by decorating the coverslip surface with F-actin binding proteins (such as inactivated myosins, or filamin) or by maintaining unanchored filaments close to the surface with a crowding agent (usually methylcellulose). Each method has its pros and cons, but for both it is not straightforward to assess whether the technique used (crowding agent or multiple anchors) has any impact on the observed behavior. The flowing solution in microfluidics can potentially alter the protein interaction kinetics, however this is easy to test since the microfluidic flows can be controlled at will, in real time. For instance, it is possible to compare the observation under continuous flow, with a situation where the flow rate is reduced to zero between imaging time points (Jégou et al. 2011b). So far, none of the reactions we have studied showed any sign of being directly affected by the flow, within the range of flow rates that we commonly use (up to several hundreds of µm/s in the vicinity of the filament). Note, however, that the flow may indirectly affect reaction kinetics via the mechanical stress it applies to the filaments (see “Various mechanical stresses can be applied to the filaments” Section).

#### Filaments appear straight and parallel to the direction of the flow, easing analysis

Filaments, anchored to the coverslip surface by one end only, align with the flow. This enables the observation of straight, parallel filaments. While the random positioning of anchoring points may lead to some filaments overlapping with their neighbors, their alignment with the flow prevents the filament crossings encountered in a classical experiment where filaments have random orientations. Straight filaments without intersections make their analysis easier, including with the assistance of software for (semi-)automatic data processing (Janco et al. 2018). This is an important aspect when large amounts of data are collected (see next point). Moreover, based on the design of the experiments, it is clear which filament end is the barbed end and which is the pointed end.

Note that the filaments, even when they appear completely straight, still fluctuate laterally and vertically. Diffraction limitations of light microscopy, combined with typical exposure times in the 100 ms time-range, average out these fluctuations and the filaments thus appear as straight segments.

#### Large populations of single filaments are imaged

An essential requirement of single filament studies (and, more generally, of single molecule studies) is the observation of large numbers of individual events. An advantage that directly comes from the previous point is the possibility to generate fields of view with a high density of filaments, which can be easily analyzed. This aspect is reinforced by the possibility to create filament populations that are homogeneous in length (by growing the filaments directly in the chamber, before starting the experiment—all done thanks to the possibility to change the protein solution bathing the filaments). Imaging in one field of view a large number of filaments with similar characteristics (length, age—i.e. nucleotide content) under the same conditions is a great asset for statistical analyses.

#### Protein concentrations are thoroughly controlled, and buffered by the flowing solution

A rather obvious, though often overlooked consequence of using a constantly incoming flow of a protein solution, is that the protein concentration seen by the surface-anchored filaments is stable. There is no depletion of reagents, like there could be in a closed chamber, because fresh solution is constantly supplied by the incoming flow. Likewise, proteins detaching from the filaments (including actin monomers themselves, if the filaments are depolymerizing) are flowed away and do not alter the local protein concentration. This aspect is particularly useful when quantifying reaction rates.

#### Rapid and sequential exposure to different protein solutions

This is perhaps the most important feature of microfluidics for the study of biochemical reactions. The possibility to rapidly (< 1 s) move the boundaries between the incoming solutions in the main channel of the flow cell allows one to sequentially expose the field of view to different solutions in a time-controlled manner.

Hence the filaments can be subjected to abrupt concentration changes, providing a well-defined “time zero” for kinetic studies under the microscope. A simple example is the instant removal of actin monomers, enabling the observation of the transition from elongation to depolymerization, thereby providing insight on the nucleotide content of the recently assembled filament region (Jégou et al. 2011b). The same strategy, using microfluidics, can be applied to microtubules (Duellberg et al. 2016).

In general, sequential exposure allows the construction of filaments with a well-controlled history before exposing them to a protein of interest. A specific and useful consequence is the possibility to build filaments with unlabeled segments (Fig. 1b). This has been instrumental in debunking an unsuspected artefact due to labeling and illumination: that fluorescently labeled actin subunits form a covalent bond with neighboring subunits over time when exposed to light (Niedermayer et al. 2012).

Another interesting use of this controlled sequential exposure is the possibility to study the binding of a fluorescently labeled protein to actin filaments, without using TIRF microscopy: by briefly exposing the filaments to an identical solution of unlabeled proteins, thereby removing the fluorescent background, filaments can be imaged in epifluorescence (Wioland et al. 2017; Cao et al. 2018). This can also be useful when exposing filaments to high concentrations of labeled proteins, and TIRF may not suffice to decrease the background signal and allow the acquisition of well-contrasted images.

#### Various mechanical stresses can be applied to the filaments

The viscous drag exerted by the flowing fluid on the actin filament can be used to apply a significant tension (up to several tens of picoNewtons) to the filament. When filaments are anchored by one end, the tension is not uniform. It increases progressively from the free end to the anchoring point, where the tension is maximum. The precise calibration of this tension requires determining the filament profile in z, i.e. its height above the surface, in the flow gradient. At low flow rates (flow gradient above the surface < 1000 s^−1^), the filament height rapidly increases from the anchor to a stable height, about 250 nm above the surface, and the tension gradient along the filament can be considered linear (Jégou et al. 2013; Wioland et al. 2019a). At stronger flow rates (flow gradient > 2000 s^−1^) the filament height remains below 250 nm and increases linearly from the anchor to the free end, making the tension gradient along the filament non-linear (Wioland et al. 2019a).

Generating such tension gradients on actin filaments can be a powerful way to directly probe a large range of local tensions. However, a nearly uniform tension can also be obtained using microfluidics: by anchoring both filament ends to the surface, and applying a flow perpendicular to the main axis of the filament (Wioland et al. 2019a). Note that, as filaments fluctuate spatially in a flow gradient, the applied tension fluctuates rapidly (typically ± 30% around its mean value) (Jégou et al. 2013). Compared to optical trapping, a powerful technique widely used to put filaments under tension, microfluidics thus is not as accurate. However, this limitation is compensated by the fact that force is simultaneously applied to hundreds of individual filaments, which can all be analyzed (see “Large populations of single filaments are imaged” Section).

Microfluidics can also be used to bend actin filaments, a configuration which is difficult to achieve with other techniques. A simple way to do so is to anchor stable filament seeds to the surface with a fixed orientation that differs from that of the flow (Wioland et al. 2019a). This can be achieved by applying different flow directions at, or near, the junction between incoming channels. Similarly, microfluidic flows have been used, in combination with micropatterning, to bend microtubules and reveal the consequences of this mechanical stress (Schaedel et al. 2015). Compared to other studies where the actin filaments, including their curved regions, are clamped to the surface (Risca et al. 2012), microfluidics-based techniques have the advantage that the bending is reversible and can be modulated by adjusting the flow rate. More sophisticated approaches using microfluidics and micropillars to deviate the flow lines, have been proposed (Jégou et al. 2011a; Carlier et al. 2014) but they are more difficult to implement and certainly limited to the application of large radii of curvature.

Finally, microfluidics can help bind filaments to fixed points, or to other filaments. These anchors constrain the filaments, in particular they reduce their ability to relax torsional stress (Wioland et al. 2019a).

#### Microfluidics can be combined with other techniques

The microfluidics configuration that we present here is very simple, and it can be extended to include more sophisticated features found in other microfluidics setups. For example, in order to observe filaments and change their chemical environment without imposing an orientation, one may wish to generate regions with no significant flow. This may be achieved by monitoring filaments in side chambers, or cavities, where the reagents will be exchanged with the main channel by diffusion (Cambier et al. 2014; Deshpande and Pfohl 2015; Wioland et al. 2019a) (Fig. 1f) or by using membranes through which proteins can diffuse (de Jong et al. 2006). Another classic development of microfluidics is the generation of protein gradients (Jeon et al. 2000), and strategies can be devised to generate static gradients with no flow in the region of interest (Strelnikova et al. 2016), some also using permeable membranes (Diao et al. 2006).

In addition, a number of techniques with no connection to microfluidics can be implemented. Micropatterns can be used to create specific regions within the chamber, for example to control where the filaments will bind to the surface in order to further ease the subsequent data analysis (an example with microtubules can be found in (Schaedel et al. 2015)). Lipid bilayers can be used in order to bind proteins and filaments to a fluid surface, rather than to a rigid glass coverslip. This technique, inspired by DNA studies, can also be applied to actin filaments (Courtemanche et al. 2013) (Suzuki et al. 2019). Moving away from the surface, an optical trap can be added to the microfluidics device (Jégou et al. 2013).

Some techniques can even be made more efficient thanks to microfluidics. An important example is the measurement of the polarization of the light emitted by a single fluorescent label, in order to assess the orientation of the actin subunit to which the label is bound. This powerful technique, introduced over two decades ago, allows the monitoring of the rotation of an actin filament around its main axis by measuring the two components of the polarized emission, at + 45° and − 45° with respect to the filament’s axis (Sase et al. 1997). To be exploited to its full potential, however, this technique requires the filaments’ main axis to point stably in a well-defined direction, which is extremely unlikely in experiments where the filaments are randomy oriented or free to wiggle around (Mizuno et al. 2011). Microfluidics, by orienting all the filaments in a fixed direction (see “Filaments appear straight and parallel to the direction of the flow, easing analysis” Section) greatly improves the throughput of the experiment: one simply needs to position the polarization filters at + 45° and − 45° with respect to the direction of the flow. As we shall see in the next sections, this combination of techniques is instrumental to study the rotation of formin-elongated filaments (Suzuki et al. 2019) and the application of a torsional moment by ADF/cofilin decorating the sides of the filaments (Wioland et al. 2019a).

## Using microfluidics to study rapid filament elongation by formins

### A brief introduction to formins

More details on formins can be found in recent reviews, focusing on physiological aspects (Bogdan et al. 2013), on their mechanistic molecular details (Courtemanche 2018) and on their role in mechanotransduction (Zimmermann and Kovar 2019).

Formins are a protein family (15 genes in humans) which plays an important role in actin assembly. They ease the nucleation of new actin filaments and, most notably, they accelerate barbed end assembly from profilin-actin. In cells, formins are responsible for generating elongated, unbranched actin filament networks, such as filopodia, stress fibers and the cytokinetic ring. They also cooperate with other ABPs to generate complex actin networks, including lamellipodia and the cell cortex. Formins thus participate in various cellular functions, including adhesion, division and motility. Malfunction of formins can lead to a number of diseases, such as neuropathies, cardiomyopathies, and kidney disease.

At the molecular level, formins are fascinating machines. Formins have rather high molecular weights (120–220 kDa) and form homodimers. They contain two key functional domains: the highly conserved Formin Homology 2 (FH2) domain is responsible for tracking the actin filament barbed end processively, while the disordered Formin Homology 1 (FH1) domain harbors several polyproline motifs which bind profilin-actin complexes in order to speed up filament barbed end assembly. Most members of the formin family are auto-inhibited, typically by having their DID (Diaphanous inhibitory) domain binding directly to their DAD (Diaphanous autoinhibitory) domain, and are activated by Rho GTPase binding to their RBD (Rho-binding) domain (Chesarone et al. 2010). Most formin studies in vitro use truncated formin constructs in order to circumvent auto-inhibition. In the following, the term “formin” will refer to these active constructs.

Studying formins’ hallmark ability to rapidly assemble long actin filaments requires distinguishing it from its other activities, such as nucleation. To do so reliably, individual filaments must be monitored. In particular, two key parameters should be measured: the elongation rate of the barbed end when it is occupied by a formin, and the rate at which the formin detaches from the barbed end (i.e. characterizing its processivity). As we shall see in the next paragraphs, microfluidics offers many advantages for such studies.

### Insights from experiments with formins interacting with the unanchored filament barbed end

**Fig. 2:**
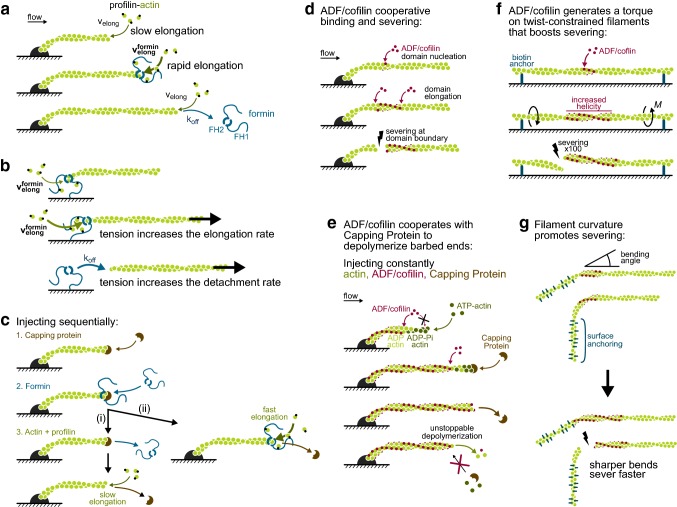
Microfluidics applied to the study of formins and ADF/cofilin. **a**–**c** Formins. **a** Formins at the barbed ends of filaments anchored by their pointed end (spectrin-actin seed) can be detected by the faster elongation rate from profilin-actin. The elongation rate is directly measured, and the formin off-rate can be also determined. **b** When formins are anchored to the surface, they can be exposed to mechanical tension (viscous drag on the filament). Filament detachment from the surface indicates formin departure from the barbed end. **c** Sequential exposure of a filament to Capping Protein, then formin, then profilin-actin, reveals that both CP and the formin can coexist at the barbed end. Different outcomes are then possible: the formin can detach first (**a**) and the filament will elongate slowly once the capping protein has also detached; or the capping protein can detach first (**b**) and the filament elongates rapidly from profilin-actin. **d**–**g** ADF/cofilin. **d** ADF/cofilin binds cooperatively to ADP-F-actin, thereby forming domains on the filaments. Severing events occur at the boundaries between ADF/cofilin domains and bare filament regions. **e** Capping a filament barbed end allows ADF/cofilin to fully decorate this filament, which ages and becomes fully ADP-actin. The barbed end of the ADF/cofilin-decorated filaments uncaps rapidly, and undergoes a nearly unstoppable depolymerization, as ATP-actin monomers and capping protein can barely bind to it. **f** Filaments regions between two anchoring points are constrained in twist, and ADF/cofilin thus applies a mechanical torque (M) as it binds. This torque dramatically enhances the severing rate at the domain boundaries. **g** Sketches illustrating the experiment where sharp bends are applied to filaments by anchoring a filament segment to the surface, and aligning the rest of the filament with the flow, in a different direction. Two filaments are depicted, and the one with the sharper bend is severed faster by ADF/cofilin

In the simple configuration where filaments are grown from anchored pointed ends (e.g. adsorbed spectrin-actin seeds, Fig. 2a), the barbed end elongation rates are easily measured. The presence of a formin at the barbed end can be assessed thanks to its enhancement of the elongation rate from profilin-actin. To assess the presence of the formin in the absence of profilin, one can alternate with a profilin-actin solution and determine if elongation is then fast or slow. For clarity, this can be done with different fluorescent labels on actin, resulting in striped filaments (Cao et al. 2018). Alternatively, one can use a fluorescently labelled formin (Shekhar et al. 2015), or switch to a configuration where the formin is anchored to the surface (“Insights from experiments with anchored formins” Section). This can be necessary when working with a slow formin, where the enhancement of elongation is mild and difficult to detect.

Formin off-rate measurements can be made without microfluidics (Kovar et al. 2006; Paul and Pollard 2008; Bombardier et al. 2015) but they are challenging and microfluidics makes them easier, thereby allowing the exploration of more biochemical conditions. Being able to easily monitor large populations of filaments (“Filaments appear straight and parallel to the direction of the flow, easing analysis” and “Large populations of single filaments are imaged” Sections) enables the measurement of formin survival fractions over time (i.e., what fraction of the filament barbed ends still bear a formin) and thus the determination of the off-rate. This is also made more efficient by the possibility to transiently expose barbed ends to relatively high (tens of nM) concentrations of formin (“Rapid and sequential exposure to different protein solutions” Section): this leads to an initial population where nearly all barbed ends bear a formin, and their evolution in the absence of formins in solution can subsequently be monitored.

Using microfluidics thus allowed us to explore the processivity of formins mDia1 and mDia2 under different biochemical conditions (Cao et al. 2018). We showed that, at a fixed profilin concentration, the formin off-rate increased with actin concentration, confirming an earlier report on yeast formin Bni1p (Paul and Pollard 2008), and we further showed that the off-rate increased with the ionic strength and decreased with the profilin concentration (Cao et al. 2018). The latter result indicates that profilin stabilizes formins at the barbed end and is consistent with the proposed formation of a “ring complex” consisting of simultaneous FH2-barbed end-profilin-FH1 contacts (Vavylonis et al. 2006).

Being able to sequentially expose the filaments to different protein solutions is also very useful for addressing the competition and synergy between formins and other end-binding proteins. The first example is that of an unexpected synergy between Spire and formin Fmn2 (Montaville et al. 2014). Spire contains four actin-binding, WH2 domains, and it can interact with actin in several ways, including by capping barbed ends. Using microfluidics, we have successively exposed filament barbed ends: (i) to Spire in order to cap them, then (ii) to formin Fmn2, and finally (iii) to a solution of profilin-actin. We then observed during step (iii) that some filaments, upon the departure of Spire, underwent a rapid elongation indicating that formin Fmn2 had been present at their barbed end since step (ii). Experiments with truncated Spire revealed that Spire directly recruited Fmn2 at the barbed end. Further quantification of the reactions, using different protein concentrations, showed that the Fmn2 on-rate was 30-fold higher for Spire-capped barbed ends than for bare barbed ends.

The second example is that of a competition which is not as fierce as expected, between capping protein (CP) and formins mDia1 and FMNL2 (Shekhar et al. 2015). Performing sequential exposure experiments similar to the one we have described for Spire, we showed that CP and formins could occupy the barbed end simultaneously (Fig. 2c). Quantifying formin on-rates in different situations revealed that the presence of CP at the barbed end, while it did not fully prevent the binding of formin mDia1, reduced its on-rate 18-fold. Likewise, the presence of a formin at the barbed end reduced the on-rate of CP. In the ternary, CP-formin-barbed end complex, CP and formin each have a higher off-rate than if they were alone at the barbed end. The existence of the CP-formin-barbed end ternary complex was also evidenced without microfluidics by single molecule fluorescence microscopy, at the same time as our study, with formins mDia1 and Daam1 (Bombardier et al. 2015). Each technique has its own advantages: using single-molecule imaging allowed Bombardier and colleagues to observe that formins could diffuse along the length of the filament, while microfluidics allowed us to explore broader protein concentration ranges. Combining microfluidics and single molecule observations would provide exciting experimental possibilities.

In both of these examples, being able to expose filament barbed ends (either bare, capped by Spire or capped by CP) to different concentrations of formins, including high concentrations, was instrumental in order to quantify the formin on-rates. Microfluidics made this possible because the formin solution contained no actin, and filaments could subsequently be exposed to a profilin-actin solution to monitor their behavior. In classical experiments, where all the proteins are present throughout, formins must be limited to very low concentrations in order to avoid the massive nucleation of new filaments in the sample.

### Insights from experiments with anchored formins

Besides allowing the application of tension to the formin (see next paragraphs), working with anchored formins offers a number of interesting possibilities. First, it allows one to monitor the nucleation of filaments by a fixed formin population and thus to directly determine the nucleation rate. Second, it provides a convenient way to assess the presence of the formin at the barbed end, regardless of its elongation rate, thereby allowing to determine the off-rate: detachment of the filament from the surface then indicates that the formin has left the barbed end (Fig. 2b). Naturally, in this case, formin detachment from the surface during the experiment must be controlled, and this can be done by renucleating filaments from the same surface to verify that formins are still present and active (Cao et al. 2018). Finally, when formins are anchored, filament elongation can be monitored using unlabeled actin, after a small filament segment with labeled actin is nucleated, as shown in Fig. 1 (Jégou et al. 2013; Cao et al. 2018). Both ends are easily localized: the pointed end is at the tip of the labeled segment and the barbed end is in the fixed location where the formin is anchored, which can be identified during the elongation of the labeled segment or immediately after.

All these measurements can be carried out with weak flow rates, applying a negligible force (< 0.1 pN) to the anchored formin. However, one of the great interests of this configuration is to apply significant forces to the formin. Doing so, we have shown that formins mDia1 (Jégou et al. 2013) and mDia2 (Cao et al. 2018) are mechanosensitive, as tensile forces of a few picoNewtons increased their elongation rates up by approximately two-fold. Others, also using microfluidics to apply tension to filaments have shown a similar behavior for yeast formin Bni1p anchored to a lipid bilayer (Courtemanche et al. 2013). We have also shown, by switching from elongating to depolymerizing conditions (i.e. with no actin in solution), that formin mDia1 could remain attached to depolymerizing barbed ends and withstand picoNewton forces (Jégou et al. 2013). An interesting consequence of this observation is that formins are able to put depolymerizing filaments under tension.

Other techniques have successfully been used to put filaments under tension and to further study formin mechanosensitivity: myosin motors, mimicking the physiological generation of force, have been used to pull on mDia2 and yeast formin Cdc12 (Zimmermann et al. 2017), and magnetic tweezers (Yu et al. 2017, 2018) as well as optical tweezers (Kubota et al. 2017) have been used to pull on mDia1. Compared to these techniques, the main advantage of microfluidics is that it allows the simultaneous application of tension to very large numbers of individual filaments, easing the collection of data (but the applied force fluctuates more, see “Various mechanical stresses can be applied to the filaments” Section). This point is reinforced by the fact that processivity is finite, and many formins let go of their filament before a reliable velocity measurement could be performed.

Sample size is particularly critical when studying formin processivity: while a few reliable measurements are enough to estimate the average filament elongation rate under a given set of conditions, determining the formin off-rate requires monitoring detachment events for a population of several tens of filaments. Using microfluidics, we were thus able to measure the impact of tension on the processivity of formins mDia1 and mDia2 (Cao et al. 2018). We found that the formin off-rate was extremely sensitive to force, as the application of a couple of picoNewtons was enough to increase the off-rate by roughly one order of magnitude. This mechanical effect on processively outweighed the biochemical effects mentioned previously (“A brief introduction to formins” Section). The impact of tension was also much stronger on the off-rate than on the elongation rate, and thus the application of tension led to a dramatic reduction in the resulting length of the formin-elongated filaments.

### From single filaments to filament bundles

So far, we have characterized the action of formins on single, independent filaments. In the cell context, actin filaments are organized in networks, and the action of regulatory proteins can be affected by the network geometry (Reymann et al. 2012; Jégou and Romet-Lemonne 2016). In the case of formins, filament bundles such as the ones that make up filopodia are a clear example. Microfluidics can also be a useful tool to generate such bundles from a few actin filaments and study how bundling affects formin activity. This can be achieved by elongating filaments from independent surface-anchored proteins and exposing them to a solution containing the bundling protein fascin to trigger the association of neighboring filaments (Fig. 1d). Alternatively, formins can be anchored to a lipid bilayer pattern. The viscous drag applied by the flow to the filaments then leads to their collection at the downstream border of the lipid bilayer, with a higher local density that can favor the formation of bundles (Fig. 1e).

As for single filament experiments, the precise control of biochemical conditions, the alignment of the filaments and the high throughput provided by microfluidics are beneficial to study filament bundles. In addition, multiple filaments joined together can take on complex forms, but the flow direction can simplify the geometry of the bundled filaments and make it easier to discern individual filaments and events, such as detachment, bending and buckling.

Performing experiments using microfluidics in different configurations, we have observed that bundling can significantly reduce the elongation rate of formin-bound barbed ends and increase the formin off-rate (Suzuki et al. 2019). Using the polarization of fluorescence to assess the rotation of a filament around its axis (see “Microfluidics can be combined with other techniques” Section and Fig. 1c), we showed that constraining the formin’s ability to rotate around its anchoring point further enhanced these effects.

## Using microfluidics to study the disassembly of actin filaments by ADF/cofilin

### A brief introduction to ADF/cofilin

More details on ADF/cofilin can be found in reviews, such as (De La Cruz 2009; Bravo-Cordero et al. 2013; Hild et al. 2014).

In cells, actin filament networks turnover, sometimes very rapidly, and controlling their disassembly is as important as controlling their nucleation and growth. A number of regulatory proteins are involved in filament disassembly, and the central player is the ADF/cofilin protein family (with 3 isoforms in mammals: cofilin-1, cofilin-2 and ADF, which behave very similarly and are hereafter collectively referred to as “ADF/cofilin”). ADF/cofilin is essential for a number of cell functions, and it is linked to several pathologies (Bamburg and Bernstein 2016; Shishkin et al. 2016).

ADF/cofilin binds actin monomers, but we will focus here on its binding to actin filaments and to the different ways it promotes their disassembly. As we shall see, the action of ADF/cofilin on actin filaments is complex, and what we summarize here results from decades of work from several labs (and we apologize for not citing all the articles that have led to this current understanding).

ADF/cofilin binds preferably to ADP-F-actin (i.e., the older regions of actin filaments) and does so in a cooperative fashion, leading to the formation of ADF/cofilin domains (De La Cruz 2005). These domains locally change the actin filament conformation: the helical pitch is reduced (with no measurable change in length) and the filament is locally more flexible, both in bending and torsion (McGough et al. 1997; Prochniewicz et al. 2005; McCullough et al. 2008). As a consequence, filaments sever at (or near) the boundaries between ADF/cofilin domains and bare filament regions (Fig. 2a) (Suarez et al. 2011). Filaments fully decorated by ADF/cofilin do not sever because they contain no domain boundaries. The (dis)assembly dynamics of the ends of decorated filaments differ from that of bare filaments. We discuss in “Quantifying ADF/cofilin’s many reactions” Section how microfluidics has enabled us to quantify and to bring insight into these different reactions.

In addition, ADF/cofilin binding and severing have been reported to be inhibited by mechanical tension applied to the actin filaments (Hayakawa et al. 2011), and there is recent theoretical (Schramm et al. 2017) and experimental (Mizuno et al. 2018) evidence that other mechanical constraints can have an impact on the action of ADF/cofilin. As we discuss in “Applying various forms of mechanical stress” section, microfluidics can also help to address these questions by providing new means to apply mechanical stress to actin filaments.

### Quantifying ADF/cofilin’s many reactions

The global severing activity of ADF/cofilin results from a number of individual, underlying reactions: nucleating a domain, expanding a domain (with on- and off-rates for ADF/cofilin within the domain and at the domain boundaries) and severing at each domain boundary (Fig. 2d). Microfluidics, by allowing us to sequentially expose the chamber to different solutions has allowed us to create situations where we could monitor these different reactions, and measure their kinetic rate constants (Wioland et al. 2017, 2019b). For example, exposing filaments saturated with unlabeled ADF/cofilin to labeled ADF/cofilin allowed us to measure the turnover of ADF/cofilin within a domain. Also, monitoring the growth of ADF/cofilin domains on straight filaments in the flow made it easy to track and quantify severing events, and we could thus measure, for the first time, the severing rate per domain.

The ease with which such experiments can be performed on large filament populations was instrumental in order to repeat these measurements at different values of pH (Wioland et al. 2019b). Measuring the rates for the different underlying reactions allowed us to show that, at lower pH, the reduced severing efficiency of ADF/cofilin does not reflect a reduction in the severing rate per domain (which is unaffected) but rather results from a faster decoration of the filaments by ADF/cofilin (making domain boundaries, where severing can happen, shorter lived because domains expand and merge faster).

The possibility to generate unlabeled segments (“Rapid and sequential exposure to different protein solutions” Section and Fig. 1b) has also allowed us to measure a number of these rates on unlabeled actin. This feature could prove essential for other proteins, such as some tropomyosins which do not appear to bind on labeled actin (Gateva et al. 2017).

Microfluidics also eased the observation and the quantification of depolymerization from ADF/cofilin-saturated filament ends. We showed that, compared to bare ADP-actin filaments, decoration by ADF/cofilin accelerated pointed end depolymerization, and slowed down barbed end depolymerization. The amplitude of this effect depends on the protein isoforms and on pH, but overall, depolymerization remains slightly faster at the barbed end than at the pointed end.

More strikingly, using microfluidics allowed us to come across two unexpected results at the barbed end (Wioland et al. 2017). First, we showed that soluble ADF/cofilin interacted directly with bare ADP-actin barbed ends and accelerated their depolymerization. It should be noted that another study using microfluidics also observed this acceleration of barbed end depolymerization, but mistakenly attributed it to the decoration of the filament sides instead of a direct interaction with barbed ends (Shekhar and Carlier 2017). This effect probably does not play a great role under physiological conditions, since it requires barbed ends to be in the ADP state.

Our second, unexpected observation is far more significant: we found that the barbed ends of ADF/cofilin-decorated filaments were in a state where they could hardly stop depolymerizing, even in the presence of monomeric actin and capping protein (Wioland et al. 2017). For instance, we could measure that the dissociation constant of capping protein at the barbed end was increased more than 600-fold by ADF/cofilin decoration. A consequence is that capping protein and ADF/cofilin synergize to depolymerize barbed ends: capping stops filament elongation and thus allows ADF/cofilin to decorate it all the way up to the barbed end (otherwise, the recently assembled barbed end region is mostly ADP-Pi-actin, preventing ADF/cofilin from binding there); once the filament is fully decorated, capping protein detaches and the barbed end depolymerizes, hardly getting capped by CP or “rescued” by ATP-G-actin (Fig. 2e). We believe that this effect is likely to cause filaments to depolymerize from both ends, including their barbed ends, in many cellular processes.

The discovery of this new barbed end state, as well as its quantitative characterization, were eased by the fact that filament fragments were flowed away, allowing us to observe populations for long periods of time. In a classical experiment without microfluidics, the accumulation of these fragments (and also, in some cases, of new filaments coming from the bulk solution containing methylcellulose) would have made it very difficult to follow individual filaments over time.

### Applying various forms of mechanical stress

As we have explained in the “Key advantages for the study of actin assembly dynamics” Section, microfluidics experiments can be performed in different configurations in order to put filaments under tension (“Various mechanical stresses can be applied to the filaments” Section). Doing so, we found that tension, up to 30 pN, had almost no effect on the binding and severing activities of ADF/cofilin (Wioland et al. 2019a). This result is in contradiction with optical trap and magnetic trap experiments which reported that mechanical tension protected filaments from the action of ADF/cofilin (Hayakawa et al. 2011), and more experiments are required to solve this discrepancy. Using microfluidics to bend filaments (“Various mechanical stresses can be applied to the filaments” Section and Fig. 2g), we have shown that increasing the local curvature increased the severing rate (Wioland et al. 2019a).

Since structural data shows that filaments regions decorated by ADF/cofilin have a reduced helical pitch, we expected that single filaments anchored by one end would be made to rotate around their axis as ADF/cofilin binds to them. Using the polarized emission of single fluorescently labeled actin subunits (“Microfluidics can be combined with other techniques” Section and Fig. 1c) we confirmed this mechanical effect: we quantified that the binding of approximately 90 ADF/cofilin molecules generated one full turn of the filament. A consequence is that filament regions that are held between two anchoring points and are thereby constrained in twist (they cannot rotate around their axis), undergo a mechanical torque as ADF/cofilin decorates them. By exposing populations of single- and double-anchored filaments to ADF/cofilin, we showed that constraining the twist had not impact on ADF/cofilin binding, but that it increased the severing rate per domain up to 100-fold (Fig. 2f) (Wioland et al. 2019a). This shows that ADF/cofilin, as it binds to twist-constrained filaments, has a mechanical effect (generating a torque) which dramatically enhances its biochemical action (severing filaments).

### From single filaments to cross-linked networks, protected from the flow

In cells, filaments are not only exposed to mechanical stress, they are also interconnected. Being connected constrains their twist, and based on our single-filament observations, we expected that it would greatly enhance their severing by ADF/cofilin. To test this, we needed to observe filament networks without exposing them to shear flow. As we have discussed, there are ways to create regions with no flow within a microfluidics system (“Microfluidics can be combined with other techniques” Section and Fig. 1f). We have used a simpler system, a T-shaped microchamber where solutions are injected manually, which nonetheless illustrates what can be gained by changing solutions with a flow without exposing the filaments to the flow. Here, dense quasi-bidimensional filament assemblies were put in a “dead end” region, next to the main channel where solutions could be flowed in. Filaments contained biotinylated actin and exposing them to a solution of neutravidin led to their cross-linking. Subsequent exposure to ADF/cofilin resulted in far more severing events when the filaments were crosslinked than when they were not connected (Wioland et al. 2019a).

## Conclusion and perspectives

The results we have summarized here were made easier, and sometimes even possible, to obtain by using microfluidics, which enabled the manipulation of individual actin filaments and their observation with optical microscopy. We believe some of the main assets of this technique are that it is easy to use, easy to implement in a non-microfluidics lab, and that it is easy to take advantage of its benefits for the study of actin regulatory processes in vitro.

To tackle future challenges, a simple feature like the ability to sequentially expose filaments to different protein solutions opens avenues for fruitful experiments. This will certainly be the case for the study of tropomyosins. The decoration of actin filaments by different tropomyosin isoforms appears to determine which ABPs are able to bind to the (Gunning et al. 2015; Meiring et al. 2018), and we still have a very limited understanding of how this essential regulatory mechanism is orchestrated. We still lack most of the basic rate constants for the binding of tropomyosin to actin. Progress with single filament experiments is certainly hindered by the fact that tropomyosins bind poorly to fluorescently-labeled actin (Gateva et al. 2017). As we have seen, microfluidics is a powerful tool to circumvent this limitation. In addition, sequential exposure to different solutions enables the construction of filaments decorated with different tropomyosin isoforms before exposing them to other ABPs.

Another challenge for the coming years is to be able to decipher regulatory mechanisms that involve several ABPs. A clear example is actin filament disassembly, where the action of ADF/cofilin now appears to be regulated by the combined action of other ABPs such as coronin, Actin Interacting Protein 1, twinfilin, and Cyclase-Associated Protein (Jansen et al. 2015; Johnston et al. 2015). In spite of recent progress, it is still unclear how these numerous partners act together, competing or synergizing, targeting the ends or the sides of the filaments. Being able to sequentially expose actin filaments to different combinations of proteins, while rapidly collecting data on large amounts of individual reactions, is an asset to address these questions.

Increasing the number of proteins in an experiment poses the additional challenge of testing a large number of combinations of protein concentrations. Microfluidics offers solutions to address this difficulty, by allowing the generation of concentration gradients which enable to simultaneously test many concentrations in one experiment. This can be coupled to gradients in the surface density of anchored proteins (themselves generated thanks to gradients in solution as well) in order to increase further the efficiency of the system.

Finally, a current challenge for future in vitro studies is to study biochemical reactions in physically relevant conditions. We have seen simple examples of how microfluidics can be a powerful tool to do so, and the bottom-up reconstitution of higher-order actin networks is now within reach thanks to the combination of microfluidics and surface patterning. In the future, these approaches should help answer more complex questions, such as, “how are ABPs sensitive to mechanical tension in a stress fiber?” Or, “which parameters affect the constriction of the cytokinetic ring?”
